# Race and Ethnicity and Prehospital Use of Opioid or Ketamine Analgesia in Acute Traumatic Injury

**DOI:** 10.1001/jamanetworkopen.2023.38070

**Published:** 2023-10-17

**Authors:** Dalton C. Brunson, Kate A. Miller, Loretta W. Matheson, Eli Carrillo

**Affiliations:** 1 School of Medicine, Stanford University School of Medicine, Stanford, California; 2 Department of Epidemiology and Population Health, Stanford University School of Medicine, Stanford, California; 3 Quantitative Sciences Unit, Stanford University School of Medicine, Stanford University, Stanford, California; 4 Department of Emergency Medicine, Stanford University School of Medicine, Stanford, California

## Abstract

**Importance:**

Racial and ethnic disparities in pain management have been characterized in many hospital-based settings. Painful traumatic injuries are a common reason for 911 activations of the EMS (emergency medical services) system.

**Objective:**

To evaluate whether, among patients treated by EMS with traumatic injuries, race and ethnicity are associated with either disparate recording of pain scores or disparate administration of analgesia when a high pain score is recorded.

**Design, Settings, and Participants:**

This cohort study included interactions from 2019 to 2021 for US patients ages 14 to 99 years who had experienced painful acute traumatic injuries and were treated and transported by an advanced life support unit following the activation of the 911 EMS system. The data were analyzed in January 2023.

**Exposures:**

Acute painful traumatic injuries including burns.

**Main Outcomes and Measures:**

Outcomes were the recording of a pain score and the administration of a nonoral opioid or ketamine.

**Results:**

The study cohort included 4 781 396 EMS activations for acute traumatic injury, with a median (IQR) patient age of 59 (35-78) years (2 497 053 female [52.2%]; 31 266 American Indian or Alaskan Native [0.7%]; 59 713 Asian [1.2%]; 742 931 Black [15.5%], 411 934 Hispanic or Latino [8.6%], 10 747 Native Hawaiian or other Pacific Islander [0.2%]; 2 764 499 White [57.8%]; 16 161 multiple races [0.3%]). The analysis showed that race and ethnicity was associated with the likelihood of having a pain score recorded. Compared with White patients, American Indian and Alaskan Native patients had the lowest adjusted odds ratio (AOR) of having a pain score recorded (AOR, 0.74; 95% CI, 0.71-0.76). Among patients for whom a high pain score was recorded (between 7 and 10 out of 10), Black patients were about half as likely to receive opioid or ketamine analgesia as White patients (AOR, 0.53; 95% CI, 0.52-0.54) despite having a pain score recorded almost as frequently as White patients.

**Conclusions and Relevance:**

In this nationwide study of patients treated by EMS for acute traumatic injuries, patients from racial or ethnic minority groups were less likely to have a pain score recorded, with Native American and Alaskan Natives the least likely to have a pain score recorded. Among patients with a high pain score, patients from racial and ethnic minority groups were also significantly less likely to receive opioid or ketamine analgesia treatment, with Black patients having the lowest adjusted odds of receiving these treatments.

## Key Points

**Question:**

Are there racial or ethnic disparities in how emergency management services professionals record and treat pain with opioid or ketamine analgesia for traumatic injuries?

**Findings:**

In this cohort study of over 4.7 million patient encounters across the US during a 3-year period (2019-2021), among patients with acute traumatic injuries, patients from minoritized racial and ethnic groups were less likely to have a pain score recorded. Among patients with a high pain score, Black patients were significantly less likely to receive analgesia when compared with White patients.

**Meaning:**

These results suggest that patients from racial and ethnic minority groups with acute traumatic injuries do not have their pain treated equitably in the prehospital setting.

## Teaser

This cohort study assesses whether racial and ethnic disparities exist in rates of emergency medical services practitioners recording a pain score and treating pain with opioid or ketamine analgesia among patients with acute traumatic injury.

## Introduction

Pain is one of the most common symptoms experienced by patients presenting to the emergency department (ED),^1^ and the association of patient race and ethnicity with the disparate management of pain in the ED has been well characterized.^2,3,4,5,6^ Given that approximately 20% of all ED patients arrive via ambulance, the initiation of appropriate pain control by paramedics is crucial.^7^ Patients can experience delays in pain control upon arrival to the ED due to the time required to triage, room, evaluate, and treat, along with other logistic hinderances.^8^ Prehospital care practitioners activated by 911 emergency services have a range of scope and practice abilities depending on geographic location and level of licensure.^9,10^ Even though protocol variations exist, the national scope of practice for emergency medical services (EMS) allows for administration of parenteral opiates and ketamine for analgesia.^11^

The National Emergency Medical Services Information System (NEMSIS) aggregates EMS data nationwide using standard data elements.^12,13^ NEMSIS has provided many useful insights into the practices of prehospital care practitioners.^14,15,16,17^ The national data set showed pain was the most likely primary symptom to be reported to EMS care practitioners, and its underlying etiology was most likely a traumatic injury, a trend that has continued over the past 10 years.^18,19^

Recent guidelines for pain management of traumatic injuries set forth by an expert panel enumerate 4 strong recommendations: (1) assess pain as part of general patient care, (2) consider all patients with acute traumatic pain to be candidates for analgesia, regardless of transport time, (3) use narcotic analgesia for moderate to severe pain, and (4) reassess patients and monitor for adverse effects.^20^ Despite the importance of prehospital pain management and known disparities by race and ethnicity in the hospital setting, most studies continue to demonstrate inequities in prehospital pain medication administration.^21,22,23,24,25,26,27,28,29,30^

Prior studies using NEMSIS data did not have the advantage of a pain score variable. To gain a snapshot of the entire US and how its agencies administer medications, we used the newly available pain score field and the robust size of the data set to expand on prior research previously limited to smaller cohorts or individual agencies. Focusing on patients with traumatic injuries, our first aim was to assess whether patients’ race and ethnicity were associated with the recording of a pain score by an EMS care practitioner. Our second aim focused on the subset of traumatic injury patients who reported a high pain score, to measure the association between a patient’s race and ethnicity and the care practitioner’s administration of opioid or ketamine analgesia.

## Methods

This study followed the Strengthening the Reporting of Observational Studies in Epidemiology (STROBE) reporting guidelines. The analysis was conducted with SAS Enterprise Guide version 8.1, update 2 (SAS Institute Inc). The prespecified statistical analysis plan was registered at Open Science Foundation.^32^ The protocol for the study and waiver of informed consent was approved by the Stanford University institutional review board (Human Subjects Review Board).

### Data Source

In NEMSIS, which currently includes data from 50 states and 4 islands or territories, each record represents an activation of an EMS asset. In addition to the publicly available data, 2 nonpublic data elements were requested from the NEMSIS Tactical Assistance Center (TAC), which administers the database. The first was demographic data from the American Communities Survey (ACS) 5-year estimates, which the TAC linked to the NEMSIS data through zip code, which was masked to the research team. The second was agency identifier, coded by TAC to maintain anonymity.

### Analytic Cohort

We selected individual activations from the years 2019 to 2021 involving patients aged 14 to 99 years located in the 50 US states and territories. We included 911 scene responses by advanced life support (ALS) paramedics who treated and transported the patient by ground, and excluded those who had been cared for by other health care practitioners, such as interfacility transfers. We restricted our cohort to acute traumatic injuries such as bone fractures or burns, as defined by *International Statistical Classification of Diseases and Related Health Problems, Tenth Revision (ICD-10)* codes S00-S99, T0-T14, T20-T32, and G89.11. We excluded activations lasting 5 minutes or less because the paramedic would not likely have had time to conduct an assessment with vital signs, which are required before administering opioids or ketamine. We excluded the few activations lasting over 4 hours as extreme outliers. Patients could have more than 1 activation if, for example, 2 units respond to a scene, and each reported 1 activation to NEMSIS. In these cases, we included only the transporting agency.

Last, we removed all activations from agencies that did not record a single pain score or administer a single dose of opioid or ketamine analgesia to any trauma patients during our study years. These agencies likely had nonclinical barriers such as written policies precluding them from giving opioids or ketamine, or agency-wide technical difficulties with reporting data to NEMSIS.

### Defining Key Variables

Pain scores were measured on a scale from 0 to 10, with 0 being no pain and 10 being the strongest pain. If multiple pain scores were recorded, the highest pain score was selected without regard to time of pain medication administration. Within the EMS scope of practice, ketamine has multiple indications including pain, induction for airway management, and altered mental status. Doses for pain are smaller than for other indications, so we only counted ketamine as analgesia if the dose was 100 mg or less. NEMSIS uses the race and ethnicity classifications set forth by the Office of Management and Budget.^31^ In this analysis, race and ethnicity were combined into 1 variable, which is referred to as race and ethnicity, even as we recognize that this approach may misclassify some patients. Race and ethnicity are required variables in the patient care reports of many states, but the method of collection is similar to other acute care health care settings in that it is comprised of a combination of self-report, observer report, or choosing unknown. Results are reported using the same categories of race and ethnicity as NEMSIS, with the exception of multiple race, which is composed of records where more than 1 race was recorded.

### Statistical Analysis

We describe the analytic cohort with counts and percentage distributions for all variables. To address the aims, we built multivariable logistic regression models estimating each binary outcome from patient characteristics (race and ethnicity, age, gender), encounter characteristics (time spent with paramedic), agency characteristics (agency trauma call volume), regional characteristics (urbanicity, census division), and year. We accounted for agency-level intraclass correlations by fitting a random effect for agency. The large number of agencies in the data set precluded a fixed effect model. The dependent variable for aim 1 was whether a pain score was recorded, and for aim 2 whether either an opioid or ketamine analgesia was administered. For aim 2, the population was restricted to patients with a high pain score (7 or higher), and the specific integer pain score was included as an additional control in the model. Results are reported as adjusted odds ratios (AORs) with 95% CIs.

The analyzed population includes greater than 1 million records for both aims, and the models contain only 8 or 9 variables. As a result of this overpower, we a priori expected that all associations would be highly significant, and the risk of false discovery would be high. Our variable of interest was the patient’s race and ethnicity; to avoid multiple testing we present *P* values and interpret the results on race and ethnicity only. We consider the other variables as controls, so we present their AOR estimates in Supplement 2, but do not present *P* values or interpret their results.

The race and ethnicity variable in NEMSIS has high missingness, which may not be at random, threatening the validity of our primary risk factor. To check our complete-case models, we ran multiple sensitivity analyses. First, noting that missingness of race and ethnicity was strongly related to census division, we grouped the divisions based on level of missingness: high (40% or more missing), medium (10% to 40% missing), and low (below 10% missing). We ran both complete-case models in each of these 3 groups and compared their AORs on the race and ethnicity variable. Second, we imputed missing race and ethnicity values using the zip code–level ACS variables: percentage male, percentage of each of 7 specific race and ethnic categories, percentage of each of 3 specific levels of educational attainment, percentage of households below the poverty line, and percentage of households who speak a language other than English. We then compared the AORs for the race and ethnicity variable for both aims on the complete case vs imputed data sets. Multiple sensitivity analyses were conducted for both aims including all patients irrespective of pain score recording or level, limiting the analysis to those agencies with less than 3% missingness, and controlling for grouped *ICD-10* codes.

## Results

**Figure 1.**
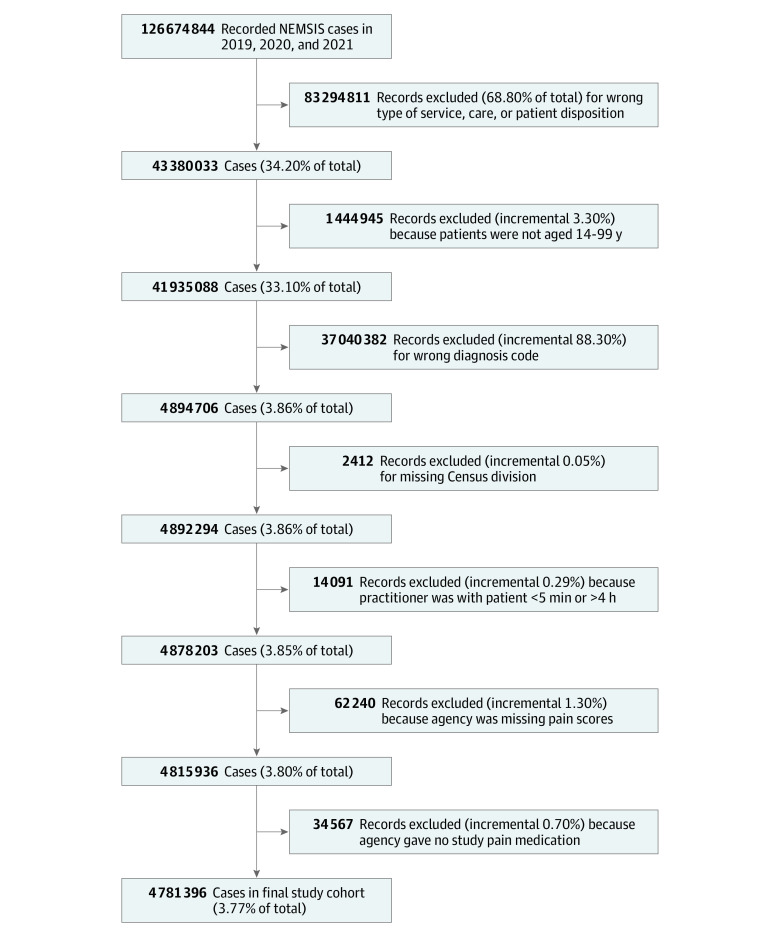
Study Flow Diagram

A total of 4 781 396 EMS activations (median [IQR] age, 59 years [35-78 years]; 2 497 053 female [52.2%]; 742 931 Black [15.5%], 411 934 Hispanic or Latino [8.6%], 2 764 499 White [57.8%]) met criteria for acute traumatic injury during the study period (Table 1; Figure 1). The smallest race and ethnicity groups were patients reporting multiple races (16 161 [0.3%]), Native Hawaiian or other Pacific Islander patients (10 747 [0.2%]), and American Indian or Alaskan Native patients (31 266 [0.7%]), each composing less than 1% of the sample. Race and ethnicity were unknown or missing in 744 145 EMS activations, or 15.6% of the data.

**Table 1.** Demographic and Clinical Characteristics of the Study Cohort

Characteristics	Patients, No. (%) (N = 4 781 396)
Race and ethnicity	
American Indian or Alaska Native	31 266 (0.7)
Asian	59 713 (1.2)
Black or African American	742 931 (15.5)
Hispanic or Latino^a^	411 934 (8.6)
Multiple races selected	16 161 (0.3)
Native Hawaiian or other Pacific Islander	10 747 (0.2)
White	2 764 499 (57.8)
Unknown	744 145 (15.6)
Gender^b^	
Female	2 497 053 (52.2)
Male	2 270 666 (47.5)
Age, y	
Continuous, median (IQR)	59 (35-78)
Categorical	
Adolescents, 14-19	266 988 (5.6)
Young adults, 20-29	593 164 (12.4)
Adults, 30-59	1 544 830 (32.3)
Senior adults, 60-74	1 312 342 (27.4)
Older adults, 75-99	1 064 072 (22.3)
Duration EMS was with patient, min	
Continuous, median (IQR)	26 (19-36)
Short time, 5 to <15	543 609 (11.4)
Moderate time, 15 to <30	2 357 033 (49.3)
Long time, 30 to <45	1 296 903 (27.1)
Extended time, 45 to 240	583 851 (12.2)
Agency size^c^	
Small (1 to <375)	1 195 339 (25)
Medium (375 to <1172)	1 193 447 (25)
Large (1173 to <4080)	1 201 671 (25)
Extra large (4080 to <33 122)	1 190 939 (25)
Urbanicity^b^	
Urban	3 946 465 (82.5)
Rural or wilderness	400 605 (8.4)
Suburban	302 939 (6.3)
Census division	
New England	177 869 (3.7)
Middle Atlantic	374 545 (7.8)
East North Central	671 689 (14.0)
West North Central	334 390 (7.0)
South Atlantic	1 328 583 (27.8)
East South Central	279 570 (5.8)
West South Central	590 115 (12.3)
Mountain	380 557 (8.0)
Pacific	644 078 (13.5)
Year	
2019	1 441 008 (30.1)
2020	1 580 382 (33.1)
2021	1 760 006 (36.8)
Pain score	
Score available	
Yes	3 276 692 (68.5)
No	1 504 704 (31.5)
Maximum pain score recorded (3 276 692 available)	
No pain (0)	446 132 (13.6)
Low to medium (1-6)	1 525 777 (46.6)
High (7-10)	1 304 783 (39.8)
Pain medication given	
Patients with high (7-10) maximum pain score (1 304 783 patients)	
Yes	479 694 (36.8)
No	825 089 (63.2)
By maximum pain score (479 694 patients)	
7	31 740 (2.4)
8	120 131 (9.2)
9	45 472 (3.5)
10	282 351 (21.6)

Abbreviation: EMS, emergency medical services.

^a^ Hispanic or Latino includes patients who reported multiple selections alongside Hispanic or Latino.

^b^ Missing or unknown not shown included under 0.3% for gender, 2.7% for urbanicity.

^c^ Agency size is defined by each agency’s average annual trauma call volume (patient records) in our study cohort.

**Figure 2.**
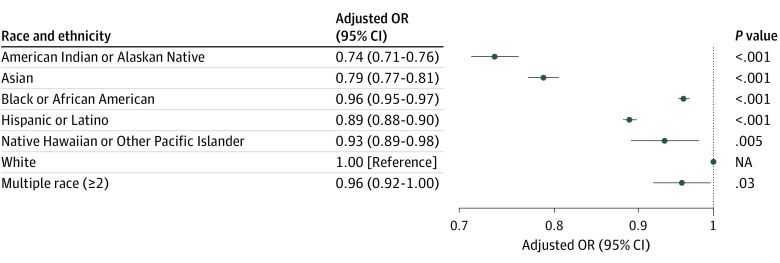
Adjusted Odds of Practitioner Recording Pain Scores by Patient Race and Ethnicity ^a^Adjusted for age, gender, region, urbanicity, agency size, year, and duration of encounter.

In this sample of acute traumatic injury primary impressions, a pain score was recorded in 3 276 692 EMS transports (68.5%) (Table 1). Adjusting for all other factors, all race and ethnicity groups had lower odds of having a pain score recorded compared with White patients (Figure 2). The largest disparities were seen in American Indian or Alaskan Native (AOR, 0.74; 95% CI, 0.71-0.76) and Asian (AOR, 0.79; 95% CI, 0.77-0.81) patients. Smaller disparities were present for other race and ethnicities such as Black (AOR, 0.96; 95% CI, 0.95-0.97). AORs for all control variables are presented in eTables 1 and 2 in the Supplement 1, but are not interpreted here.

**Figure 3.**
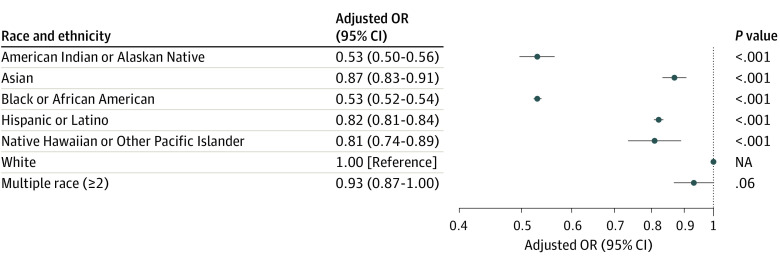
Adjusted Odds of Analgesia Administration for Patients With High Pain Scores by Race and Ethnicity ^a^Adjusted for age, gender, region, urbanicity, agency size, year, duration of encounter, and pain score.

Among patients with a pain score recorded, 1 304 783 (39.8%) reported high pain of 7 or greater, and among those with high pain, 479 694 (36.8%) received opioid or ketamine analgesia (Table 1). In the multivariable model, all racial and ethnic minority groups were less likely to receive pain medication compared with White patients, even in the presence of high pain and adjusting for all other factors (Figure 3). Black patients (AOR, 0.53; 95% CI, 0.52-0.54) and American Indian and Alaskan Native patients (AOR, 0.53; 95% CI, 0.50-0.56) were only about half as likely as White patients to receive pain medication. Patients from other racial and ethnic groups were between 7% and 20% less likely than White patients to receive pain medication.

The sensitivity analysis results are presented and discussed in depth in the eMethods in Supplement 2 and summarized here. Missingness of the race and ethnicity data varied strongly by census division. Divisions with low missingness were centered in the South, ranging from West South Central (24 098 [4.1%]) to the South Atlantic (78 420 [6.0%]) (Table 2). Moderate missing divisions were in the Midwest and West and ranged from East North Central (101 717 [15.1%]) to Pacific (142 120 [22.1%]). High missing divisions (over 40%) were in the Northeast (Middle Atlantic, 160 277 [42.7%]; New England, 86 797 [48.7%]).

**Table 2.** Missing Race and Ethnicity by Census Division

Category	Census division	Total, No.	Missing race, No. (%)
High missing	New England	177 869	86 797 (48.7)
	Middle Atlantic	374 545	160 277 (42.7)
Moderate missing	Pacific	644 078	142 120 (22.1)
	Mountain	380 557	80 804 (21.2)
	West North Central	334 390	54 571 (16.3)
	East North Central	671 689	101 717 (15.1)
Low missing	South Atlantic	1 328 583	78 420 (6.0)
	East South Central	279 570	15 341 (5.5)
	West South Central	590 115	24 098 (4.1)

Models for each aim were run based on the level of missingness. For aim 1, the 95% CIs arising from the low-, moderate-, and high-missingness regions of the country all overlapped, suggesting that the missingness on race and ethnicity may not have been systematically related to census division. The exception was the American Indian and Alaskan Native group, which had one of the smallest samples, especially in the high missingness regions (Northeast). The AOR estimates using imputed race and ethnicity were similar to the complete case estimates for all race and ethnicity groups. For American Indian and Alaska Native patients, this suggests that the differences noted in the first sensitivity analysis were due to small sample size and not differential missingness (eFigure 1 in Supplement 2).

For aim 2, the samples were smaller. For American Indian and Alaskan Native and the Hispanic and Latino groups, notable differences exist in AOR estimates between the low-, moderate-, and high-missing areas, suggesting that the race and ethnicity data may not have been missing at random. When using imputed race, all estimates fell within the 95% CI of the complete case analysis except with Black and African American patients. This suggests that there might exist some bias in recording of race and ethnicity for these patients, but it is not of great magnitude given the small absolute difference of the values (eFigure 2 in Supplement 2). Of the multiple sensitivity analysis conducted, no sensitivity analysis showed a change in trend from the complete case analysis and differences in point estimates were not of significant difference (eTable 3; eTable 4 in Supplement 2).

## Discussion

Building on an improved NEMSIS database that contains pain scores, we were able to better control for variables that might contribute to treatment disparities by race and ethnicity. In order to reduce the bias that is introduced in the clinical care of subjectively painful conditions, we limited our study to acute traumatic injury primary impressions. This may bias our results toward the null, as racial and ethnic disparities seem to be less apparent in the ED setting when fractures^33^ and conditions more easily identified as painful are present (burns, open fractures, high-energy mechanisms).^34,35^

In aim 1, we found that pain scores were not recorded for 31.5% of patients who had an acute traumatic injury primary impression. Any patient who experiences a traumatic injury should have a pain score obtained and recorded as part of general patient care per the National Association of EMS Physicians (NAEMSP) evidence-based guidelines. We further found that pain scores were less likely to be recorded for all race and ethnicity groups compared with White patients, and that these disparities were largest for Asian patients and American Indian or Alaskan Native patients. For patients reporting multiple races, the evidence for a difference from White patients was marginal.

In aim 2, we found that 63.2% of patients reporting high pain were not administered opioids or ketamine analgesia (Table 1), which is also inconsistent with recommended guidelines for pain management. Again, we found evidence of disparities by race and ethnicity, with lower adjusted odds of pain medication administration among all groups compared with White patients, with marginal evidence for a disparity among multiple race patients. Here, the largest observed disparities were among Black patients and American Indian or Alaskan Native patients.

Comparing results across the 2 aims, we note that American Indian or Alaskan Native patients appear to be most disadvantaged relative to White patients, both in recording pain scores and in receiving pain medication. This may be due to a multitude of factors, including racial bias, but also statistical bias in that they comprise a small percentage of the US population and may be clustered in areas that have low rates of recording race in traumatic injury encounters.

Black patients are nearly as likely as White patients to have a pain score recorded (AOR, 0.96), yet when they report high pain, they are much less likely to receive pain medication (AOR, 0.53). Of all racial and ethnic groups, this is the largest difference between the 2 models. This large difference indicates that among certain patient populations with severe pain, evaluating and recording pain level is not enough to ensure administration of analgesia.

The causes of the disparities we observed in pain score recording and analgesia administration unfortunately cannot be fully understood from the NEMSIS data. In speculating about potential causes, we recognize that racial and ethnic categories are constructs without biological meaning, and that the stubborn disparities by race and ethnicity reflect complex social dynamics. One possible dynamic is the opioid epidemic, which can cause people both to decline opioids out of fear of potential drug addiction and, conversely, to inflate their pain score to increase the chances of receiving opioids. This may be at work in different ways across the country and by racial and ethnic groups.^36^ Another dynamic is the norms and policies of the various states and transporting agencies regarding data collection, data quality, and reporting to NEMSIS. We do note differential recording of patient race and ethnicity by census region, which may drive or distort some of the observed associations. Finally, implicit or explicit bias among EMS practitioners toward various racial and ethnic groups can promote disparities.^37^ Research has shown that the level of implicit racial bias among health care professionals roughly mirrors that of the wider population.^38^

### Limitations

This study had limitations. The biggest threat to validity, in our view, is that 15.6% of cases in our analytic sample were missing race and ethnicity, which is the central variable of interest. The sensitivity analyses evaluating this did find some evidence of missingness not at random for some race and ethnicity groups in certain analyses. Our results must be interpreted accordingly.

Second, some patient encounters could result in more than 1 activation in the NEMSIS data; for example, if both an ALS-equipped fire apparatus and a transporting agency arrived to a single patient. In that case, the transporting agency record was selected, but there is no way of knowing if pain medications were given by the other unit excluded from the analysis. We mitigated this issue in aim 2 by selecting only patients with a high pain score, meaning that any initial pain medication given elsewhere had not been effective and would likely require redosing.

Third, certain variables in NEMSIS are not required for entry and their missingness can vary drastically. For this reason, we were not able to control for certain confounders such as insurance status, which have been shown elsewhere to be related to prehospital pain care.^21^ Fourth, the NEMSIS data faces all the complexities of categorizing race and ethnicity as does all social science research on US populations. We recognize certain distortions, such as the inclusion of Hispanic and Latino patients, who could also self-report other races, as solely Hispanic and Latino in the data set. The NEMSIS data cannot ascertain whether the race and ethnicity of patients was self-identified or simply perceived by EMS practitioners. Lastly, as in all observational research, we can only detect associations, not causal relationships.

## Conclusions

This study found disparities by the patient’s race and ethnicity in prehospital care for pain due to acute traumatic injury, including in how emergency care practitioners document pain and how they administer pain medication when high pain is reported. Compared with White patients, all other race and ethnicity groups had lower adjusted odds on both outcomes, with the largest discrepancies observed among American Indian or Alaskan Native patients. Black patients were nearly as likely as White patients to have a pain score recorded, but when they reported high pain, they were much less likely to receive pain medication. The causes for these racial and ethnic disparities require further research. Subsequent efforts to measure and improve prehospital patient-centered outcomes would benefit from national and state efforts to include race and ethnicity data collection in all future patient care reports.

## Notes

Supplement 1. **eTable 1.** OR and AOR of Control Variables for Aim 1: Pain Score Recording **eTable 2.** OR and AOR of Control Variables for Aim 2: Medication Administration **eTable 3.** Sensitivity Analysis Aim 1: Pain Score Recording **eTable 4.** Sensitivity Analysis Aim 2: Medication Administration

Supplement 2.
eMethods.
**eFigure 1.** Sensitivity Analyses for Recording a Pain Score, by Race and Ethnicity of Patient **eFigure 2.** Sensitivity Analyses for Administration of Pain Medication, by Race and Ethnicity of Patient

Supplement 3.
Data Sharing Statement
