# Supplementary material for: Race and Ethnicity and Prehospital Use of Opioid or Ketamine Analgesia in Acute Traumatic Injury
Source: JAMA Netw Open. 2023 Oct 17;6(10):e2338070. doi: 10.1001/jamanetworkopen.2023.38070 (PMC10582796; doi:10.1001/jamanetworkopen.2023.38070)
Supplement: Supplement 2. — eMethods. eFigure 1. Sensitivity Analyses for Recording a Pain Score, by Race and Ethnicity of Patient eFigure 2. Sensitivity Analyses for Administration of Pain Medication, by Race and Ethnicity of Patient [file jamanetwopen-e2338070-s002.pdf]

Supplemental Online Content

Brunson DC, Miller KA, Matheson LW, Carrillo E. Race and ethnicity and prehospital use of opioid or ketamine analgesia in acute traumatic injury. *JAMA Netw Open*. 2023;6(10):e2338070. doi:10.1001/jamanetworkopen.2023.38070

eMethods.

eFigure 1. Sensitivity Analyses for Recording a Pain Score, by Race and Ethnicity of Patient

eFigure 2. Sensitivity Analyses for Administration of Pain Medication, by Race and Ethnicity of Patient

This supplemental material has been provided by the authors to give readers additional information about their work.

© 2023 Brunson DC et al. *JAMA Network Open.*

eMethods.
Aim 1: Pain score recorded

Both sensitivity analyses are motivated by the high rates of missing data on race/ethnicity, which is our predictor of interest. Supplemental figure 1 shows the adjusted ORs and CIs from both sensitivity analyses, with the primary analysis results for comparison.

Sensitivity analysis 1: Sub groups by level of missingness

For this test, we grouped the census regions into low, medium, and high level of missingness on the race/ethnicity variable. If the missingness on race/ethnicity is at random overall, then we would expect the OR estimates for all three regions to be similar. If the estimate from the high missingness region is very different from the others, then the missingness may not be at random and the main estimate may be biased.

These estimates appear in the shaded blue areas on the graph. For nearly all racial/ethnic groups, the three estimates are within the CIs of each other, and the main estimate (in orange) is within their CIs as well. This suggests that any bias due to differential missingness is small for those groups. The exception is the American Indian or Alaskan Native group. In this case, the OR estimates are quite different and no CIs overlap with each other or the main result. In the high missingness regions, the estimate is even significantly above 1, which does not seem plausible. We find this result to be likely driven by sample size, as the high missingness group includes only 463 American Indian or Alaskan Native patients (1.5% of all patients in this race/ethnicity category). However, the other two estimates, from the high- and medium- missing regions are each based on over 15,000 patients. Alaska itself falls in the medium missing category. This suggests some distortion of these estimates, and that the main results for the American Indian or Alaskan Native patients may be biased by missingness. They should be interpreted with caution.

Sensitivity analysis 2: Imputed values

For all groups, the OR estimate using the imputed race data is close to the primary analysis result and well within its 95% CI (compare orange vs. yellow series). This suggests that any bias due to differential missingness on race is small. In the case of the American Indian or Alaskan Native group, the similarity of the imputed and complete-case results shores up the conclusion that the highly varying results in sensitivity analysis 1 were due to sample size constraints, not differential missingness.

Aim 2: Pain medications given

These models are restricted only to patients whose pain score was recorded, and for whom the score was 7 or higher. The sample size of all race/ethnicity groups are accordingly smaller.

Sensitivity analysis 1: Sub groups by level of missingness

In this subgroup analysis, the samples in certain race/ethnicity groups are small. In the high missingness category (New England and Middle Atlantic states), the sample includes 646 Asian patients, 331 Multi-race patients, 124 American Indian or Alaskan Native patients, and only 83 Native Hawaiian or Other Pacific Islander patients. In the multivariable context, these sample have wider CIs than other race/ethnicity groups.

© 2023 Brunson DC et al. *JAMA Network Open.*

Figure 2 shows that for most race/ethnicity groups, the CIs for all three missingness categories (shaded in blue) overlap with each other and with the main estimate (in orange). However, significant deviations are evident for Hispanic/Latino patients: In low missingness regions, the OR is slightly higher than the main estimate, and in medium missingness regions, it is slightly lower. The estimate from the high missingness region is similar to the main estimate. If these results are true, they suggest that a bias in recording race/ethnicity exists in one direction in the low missingness regions, the opposite direction in the medium missingness areas, and not at all in low missingness regions. This does not seem plausible. Instead, we note that for Hispanic/Latino patients, the missing data may not be at random, and the estimate may be subject to bias.

For American Indian or Alaskan Native patients, the estimate for the low missingness regions is above 1, and the wide CI does not include the main estimate. As noted above, this sample consists of only 124 patients, so we assume that this estimate is unstable and reflects the small sample. Since the other two estimates from low and medium missingness areas are close to the main estimate, we do not suspect a missingness bias for this group.

Sensitivity analysis 2: Imputed values

For all but one group, the OR estimate from the imputed race data (in yellow) is close to the main complete case estimate (in orange), and within its 95% CI. For Black patients, the CIs between these estimates do not overlap: OR of 0.53 in the complete case analysis and 0.62 in the imputed analysis. This suggests that there may be some bias in recording of race/ethnicity for Black patients, but it is not of great magnitude, as the imputed estimate is only 0.09 points higher than the main estimate.

© 2023 Brunson DC et al. *JAMA Network Open.*

eFigure 1: Sensitivity analyses for recording a pain score (Aim 1), by race/ethnicity of patient

Race and Ethnicity	Adjusted OR (95% CI)[a] Primary analysis Complete case	Sensitivity analysis 1 Census divisions grouped by missingness of race/ethnicity: High missingness	Medium missingness	Low missingness	Sensitivity analysis 2 Imputed race/ethnicity
White	1.0 (reference)	1.0 (reference)	1.0 (reference)	1.0 (reference)	1.0 (reference)
Black or African American	0.96 (0.95 - 0.97)	0.94 (0.91 - 0.97)	0.95 (0.93 - 0.97)	0.96 (0.95 - 0.97)	0.95 (0.95 - 0.96)
Hispanic or Latino	0.89 (0.88 - 0.9)	0.87 (0.84 - 0.9)	0.89 (0.88 - 0.91)	0.89 (0.88 - 0.9)	0.91 (0.90 - 0.92)
Asian	0.79 (0.77 - 0.81)	0.71 (0.65 - 0.78)	0.78 (0.76 - 0.81)	0.8 (0.78 - 0.83)	0.81 (0.79 - 0.82)
American Indian or Alaskan Native	0.74 (0.71 - 0.76)	1.35 (1.05 - 1.75)	0.66 (0.63 - 0.69)	0.84 (0.8 - 0.89)	0.74 (0.72 - 0.77)
Native Hawaiian or Other Pacific Islander	0.93 (0.89 - 0.98)	0.96 (0.72 - 1.27)	0.9 (0.83 - 0.97)	0.96 (0.9 - 1.02)	0.94 (0.90 - 0.99)
Multiple race (2+)	0.96 (0.92 - 1)	0.89 (0.79 - 1.01)	1.01 (0.92 - 1.12)	0.96 (0.91 - 1)	0.97 (0.94 - 1.01)

[a] Adjusted for age, gender, region, urbanicity, agency size, year, and duration of encounter.

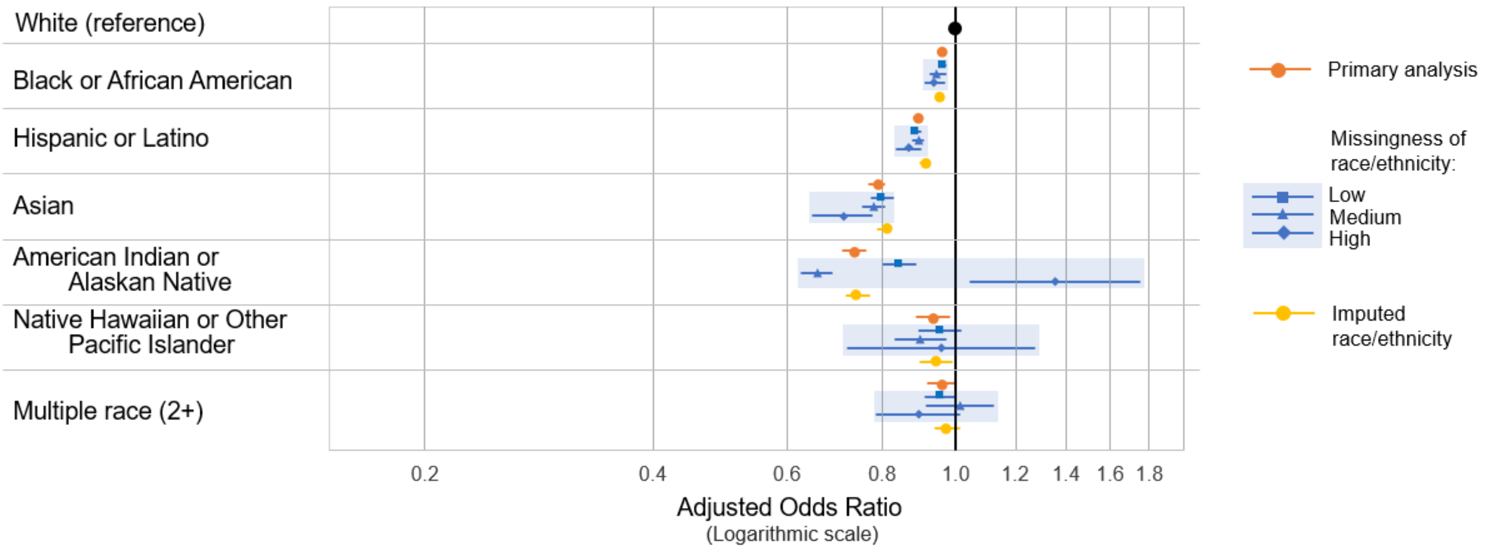

© 2023 Brunson DC et al. *JAMA Network Open.*

eFigure2: Sensitivity analyses for administration of pain medication (aim 2, by race/ethnicity of patient

Race and Ethnicity	Adjusted OR (95% CI)[a]				
	**Primary analysis** Complete case	**Sensitivity analysis 1** Census divisions grouped by missingness of race/ethnicity			**Sensitivity analysis 2** Imputed race/ethnicity
		High missingness	Medium missiningess	Low missingness	
White	1.0 (reference)	1.0 (reference)	1.0 (reference)	1.0 (reference)	1.0 (reference)
Black or African American	0.53 (0.52 - 0.54)	0.55 (0.51 - 0.59)	0.54 (0.52 - 0.56)	0.53 (0.52 - 0.54)	0.62 (0.61 - 0.63)
Hispanic or Latino	0.82 (0.81 - 0.84)	0.84 (0.77 - 0.91)	0.77 (0.75 - 0.79)	0.87 (0.85 - 0.89)	0.85 (0.83 - 0.86)
Asian	0.87 (0.83 - 0.91)	0.96 (0.74 - 1.24)	0.84 (0.79 - 0.89)	0.87 (0.82 - 0.93)	0.88 (0.85 - 0.92)
American Indian or Alaskan Native	0.53 (0.50 - 0.56)	1.11 (0.63 - 1.95)	0.5 (0.46 - 0.55)	0.58 (0.53 - 0.63)	0.55 (0.52 - 0.59)
Native Hawaiian or Other Pacific Islander	0.81 (0.74 - 0.89)	0.44 (0.20 – 1.00)	0.75 (0.66 - 0.86)	0.91 (0.78 - 1.05)	0.83 (0.76 - 0.91)
Multiple race (2+)	0.93 (0.87 – 1.00)	0.95 (0.72 - 1.27)	0.82 (0.69 - 0.97)	0.96 (0.88 - 1.04)	0.96 (0.89 - 1.03)

[a] Adjusted for age, gender, region, urbanicity, agency size, year, duration of encounter, and pain score.

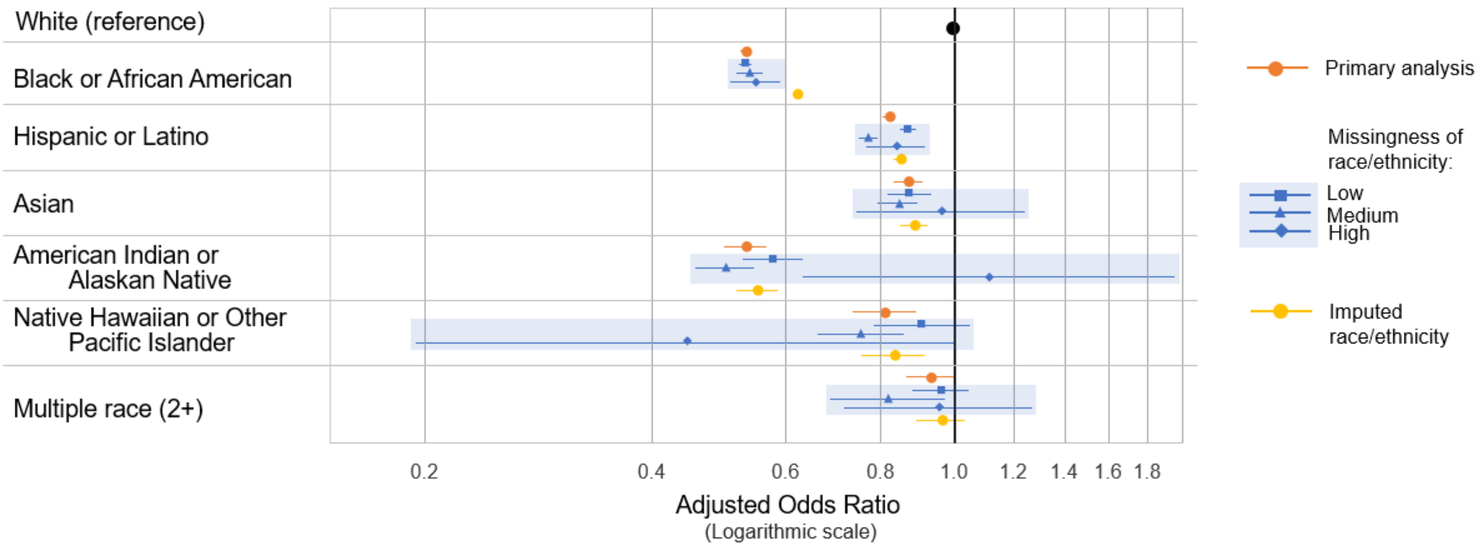

© 2023 Brunson DC et al. *JAMA Network Open.*
