# Supplementary material for: Race and Ethnicity and Prehospital Use of Opioid or Ketamine Analgesia in Acute Traumatic Injury
Source: JAMA Netw Open. 2023 Oct 17;6(10):e2338070. doi: 10.1001/jamanetworkopen.2023.38070 (PMC10582796; doi:10.1001/jamanetworkopen.2023.38070)
Supplement: Supplement 3. — Data Sharing Statement [file jamanetwopen-e2338070-s003.pdf]

Data Sharing Statement

Brunson. Race and Ethnicity and Prehospital Use of Opioid or Ketamine Analgesia in Acute Traumatic Injury. *JAMA Netw Open*. Published October 17, 2023.
doi:10.1001/jamanetworkopen.2023.38070

Data
Data available: No

Additional Information
Explanation for why data not available: Data utilized was from the National EMS Information System. Our study utilized both public and coded non-public data elements. Our team is bound by the data use agreement between Stanford University and the NEMSIS administrators. Data can be requested directly from NEMSIS, but cannot be made available from the research team. The data use agreement has been supplied in our original file upload.
